# Characterisation and optimisation of microbial production of transglutaminase produced by *Streptoverticillium cinnamoneum*

**DOI:** 10.1007/s00253-025-13606-y

**Published:** 2025-10-16

**Authors:** Vitaliy Kolotylo, Alicja Synowiec, Kamil Piwowarek, Iwona Gientka, Marek Kieliszek

**Affiliations:** Department of Food Biotechnology and Microbiology, Institute of Food Sciences, Warsaw University of Life Sciences—SGGW, Nowoursynowska 159 C, 02-776 Warsaw, Poland

**Keywords:** Microbial transglutaminase, MTG, Optimisation, RSM, *Streptoverticillium cinnamoneum*

## Abstract

**Abstract:**

Optimal conditions for the production of microbial transglutaminase (MTG) by *Streptoverticillium cinnamoneum* KKP 1648 under bioreactor conditions were determined using response surface methodology (RSM). The test medium contained soluble starch, Aminobak, waste corn steep liquor, yeast extract, and mineral salts such as KH_2_PO_4_, Na_2_HPO_4_∙12H_2_O, and MgSO_2_∙7H_2_O. The culture was conducted at 28 °C, stirring at 200 rpm, using aeration with compressed air at a flow rate of 2.0 vvm. Optimal culture parameters included a pH of 6.5, a nitrogen dose of 2.75%, and an incubation time of 72 h, and yielded MTG activities of 4.29 ± 0.08 and 22 ± 0.05 U/mL after precipitation with ammonium sulfate. The molecular weight of the enzyme was estimated to be 43 kDa by SDS-PAGE. The transglutaminase tested showed optimal activity in the temperature range 20–37 °C, with a significant decrease in activity above 50 °C and complete inactivation at 60 °C. The enzyme was stable in the pH range of 5.0–9.0, reaching maximum activity at pH 7.0. The use of waste corn steep liquor as a nutrient solution revealed a potential management strategy for this type of waste. The results suggest potential applications of transglutaminase in the food industry, particularly in enhancing the structural properties of meat, bakery, and dairy products.

**Key points:**

- *High MTG activity after 72 h of culture confirms the effectiveness of selected parameters*
- *The use of corn waste in MTG production can reduce production costs*
- *The use of the RSM method allowed us to determine the best parameters for MTG production*.
- *The MTG enzyme was stable at pH 5.0 to 9.0, with maximum activity at pH 7.0*.

**Supplementary Information:**

The online version contains supplementary material available at 10.1007/s00253-025-13606-y.

## Introduction

Microbial transglutaminase (MTG) is one of the key enzymes used in the food industry. It enables the modification of proteins by creating isopeptide bonds between glutamine (C_5_H_10_N_2_O_3_) and lysine (C_6_H_14_N_2_O_2_) residues, which leads to the formation of stable, internal or external cross-linked protein structures (Vasić et al. 2024). Thanks to these properties, MTG significantly influences texture, increases gelling, emulsifying, and foaming properties, and improves the water retention capacity of food products (Kolotylo et al. 2023). In the production of cheese and ice cream, the use of transglutaminase allows for a reduction in the addition of fats and stabilisers, which in turn reduces production costs (Taghi Gharibzahedi et al. 2018). Transglutaminase is widely used in the production of meat (Akbari et al. 2021), pasta (including the gluten-free cereal milling industry) (Ramos et al. 2023), dairy products (Taghi Gharibzahedi et al. 2018), bread (Ceresino et al. 2018), and fish (Tokay et al. 2021), where it helps to improve the quality and shelf life of the products. Transglutaminase has been used in the reuse of food waste (defatted flour and peptides from walnut and pumpkin seeds) in the production of new value-added food products (Marulo et al. 2022). In addition, producing microcapsules or edible films with MTG allows for new biodegradable packaging materials. These approaches not only reduce plastic waste, but also help to utilise food waste that would otherwise be thrown away.

Microbial transglutaminase is used not only in the food industry but also in medicine (Kim and Keillor 2020) and biomaterial engineering (Besser et al. 2020). In biotechnology, this enzyme is used for protein modification and biomaterial production as well as in research on gene therapies (Zilda et al. 2017). Li et al (2016) used transglutaminase to microencapsulate *Lactobacillus rhamnosus* GG by cross-linking the soy isolate and subsequent freeze-drying, which resulted in a high microencapsulation yield (67.4%). In a study by Heidebach et al. (2009), commercial probiotic bacteria strains showed a high microencapsulation efficiency with transglutaminase cross-linked casein gel, which ranged from 70% (for *Lactobacillus paracasei* ssp. *paracasei* F19) to 93% (for *Bifidobacterium lactis* Bb12). Thanks to the development of new research techniques, it is possible to learn more about the potential of the enzyme, which leads to the discovery of new areas of its application in industry and science (Kolotylo et al. 2023).

Transglutaminase was initially isolated from animal tissue (Keillor et al. 2014), but due to the challenges associated with its extraction and ethical concerns, increasing attention has been paid to the microbial variant of this enzyme. Unlike the animal-based enzyme, the activation of microbial transglutaminase does not require the presence of calcium ions (Kieliszek and Misiewicz 2014), which makes it a more versatile tool in many industrial processes (Vasić et al. 2023). The first reports on the microbiological sources of MTG date back to the 1980 s, when enzymes produced by microorganisms of the genus *Streptoverticillium*, especially *Streptoverticillium mobaraense*, were identified (Ando et al. 1989; Akbari et al. 2021). Thanks to its unique properties and wide range of applications, microbial transglutaminase is increasingly in demand in the food industry.

The introduction of industrial enzymes into production processes has brought significant savings by reducing the consumption of energy, water, and raw materials, as well as reducing greenhouse gas emissions (Roy Choudhury 2020). Compared to chemical modifications, enzymes offer advantages such as high specificity of the reaction and minimisation of side reactions (Vasić et al. 2023). Thanks to these properties, enzymatic protein modification is particularly effective and widely used, especially in the food industry, where precise action and resource savings are important (Keenan et al. 2021). It is therefore no surprise that this market is growing rapidly—in 2020, the global value of the food enzymes market was $2.3 billion, with the MTG segment reaching a value of around $200 million (Wu et al. 2020). Forecasts suggest that this market will exceed $3.3 billion by 2026, and the total enzyme market, including the food, biofuels, agricultural, detergent, textile, and cosmetics industries, will reach $14.9 billion by 2027 (Wu et al. 2020), which indicates a steady increase in the demand for enzymes in food processing and beyond (Kolotylo et al. 2023). Parameters such as the ability to improve protein structure and production efficiency make MTG one of the key tools used in advanced food technologies (Zilda et al. 2017).

Enzymes on an industrial scale are mainly produced through aerobic fermentation using microorganisms. The species used for the microbiological production of transglutaminase include *Streptomyces mobaraensis*, *Streptoverticillium cinnamoneum*, *Streptoverticillium lydicus*, *Streptoverticillium ladakanum*, *Bacillus subtilis*, and *Bacillus spherules* (Ceresino et al. 2018; Akbari et al. 2021; Kolotylo et al. 2024a).

Industrial waste is used as a component of microbiological media to produce enzymes, such as corn steep liquor (Kolotylo et al. 2023), potato juice water (Bzducha-Wróbel et al. 2018), glycerol fractions (Kot et al. 2020), cane molasses (Preichardt et al. 2019), brewer’s spent grain (Bianco et al. 2020), and fruit pomace (Bellucci et al. 2021). Guerra-Rodríguez and Vázquez (2014) developed a medium for the microbial production of transglutaminase (for the strain *Streptomyces mobaraensis* CECT 3230) consisting only of skimmed milk (600 g/L), waste potatoes (40 g/L), and glycerol (5 g/L). After 72 h of cultivation, they obtained an average MTG activity of 2.95 ± 0.3 U/mL, and the calculated economic yield of production was €8.11 ($9.25) of transglutaminase obtained from €1 spent on substrate ingredients (economic yield of €8.11 of MTG/€ of nutrients) (Guerra-Rodríguez and Vázquez 2014).

In the first phase of enzyme isolation, the microorganism cells are removed using methods such as centrifugation, ultrasound, or filtration to eliminate insoluble components. It is important to note that most industrial enzymes are extracellular, meaning that they are secreted into the culture medium by the cells. The enzymes are then concentrated and purified by membrane filtration or ion exchange chromatography. Depending on market requirements, different types of enzymes are available in crystalline (granular) form or in solution (Niyonzima et al. 2020; Fasim et al. 2021).

Modern optimisation of the fermentation medium has become indispensable for the efficient production of enzymes on an industrial scale. The traditionally used one-factor-at-a-time (OFAT) method, in which the ingredients are tested individually, has proven to be time-consuming and error-prone (Di Masi et al. 2024). Modern approaches based on advanced statistical and mathematical tools have significantly accelerated this process, enabling maximisation of efficiency while reducing the number of experiments (Singh et al. 2017).

Researchers now use multivariate optimisation models such as Plackett-Burman (Ma et al. 2016), Taguchi (Freddi and Salmon 2019), central composite design (CCD) (Bayuo et al. 2020), and Box-Behnken (Kolotylo et al. 2024b), which allow for the screening of key factors and the analysis of their interactions. The introduction of methods such as response surface methodology (RSM) (Ma et al. 2016; Bayuo et al. 2020; Kolotylo et al. 2024b), artificial neural networks (ANN) (Agatonovic-Kustrin and Beresford 2000), and genetic algorithms (GA) (Al-Saadi and Al-Jabri 2020) has enabled the prediction of optimal conditions by combining variables such as pH, temperature, humidity, carbon and nitrogen sources, and mineral proportions. These methods reduce optimisation time and provide accurate predictions of the effectiveness of different configurations of environmental conditions and ingredients. RSM is one of the most widely used methods, as evidenced by its effectiveness in optimising biotechnological parameters (Fasim et al. 2021).

Response surface methodology (RSM) is widely used in biotechnology to optimise the production processes of various enzymes and has remained a current and valued tool over the years. For example, using RSM made it possible to increase the yield of protease produced by the *Bacillus subtilis* K-5 strain by optimising fermentation conditions such as temperature, pH, and substrate moisture (Shad et al. 2024). Similarly, in the case of lipase produced by *Candida rugosa* NCIM 3462, RSM allowed the composition of a medium containing cheese whey to be optimised, resulting in increased enzyme activity (Rajendran and Thangavelu 2013). In another study, using RSM for the production of protease by *Bacillus subtilis* GCU-8 made it possible to identify key factors affecting the enzyme’s efficiency, which enabled precise regulation of fermentation process parameters (Adnan et al. 2014). Another example is the synthesis of α-amylase by *Trichoderma virens* DMS1963, where the use of RSM optimised the conditions for solid-state fermentation using watermelon peels as a substrate, which significantly improved the enzyme production yield (Abdel-Mageed et al. 2022). These examples emphasise the versatility and effectiveness of RSM in improving biotechnological processes related to the production of various enzymes.

In conclusion, microbial transglutaminase is a key biotechnological enzyme with a wide range of applications, both in the food industry and in other fields (Duarte et al. 2019). Thanks to advanced fermentation methods and optimised production conditions, it is possible to achieve high enzyme yields at relatively low costs, which is achieved, among other things, through the use of waste components of microbiological substrates. The use of these cheap and easily accessible raw materials not only lowers production costs but also contributes to sustainable development by reducing industrial waste. This approach supports the concept of the circular economy while increasing the economic efficiency of biotechnological processes. Further research into transglutaminase and its commercialisation potential is an important step towards industrial development and improving the quality of food products on a wider scale.

The aim of this study was to obtain a microbiological transglutaminase (MTG) preparation with high enzymatic activity using a bioreactor culture of the *Streptoverticillium cinnamoneum* KKP 1658 strain. The research aimed to optimise fermentation conditions such as pH, temperature, medium composition, and nitrogen sources in order to increase the efficiency of the enzyme production process. In addition, the biochemical properties of MTG were determined, including optimal temperature, thermostability, and pH stability, which allows for a better understanding of the enzyme’s application potential in the biotechnology industry.

## Material and methodology

### Biological material

The *Streptoverticillium cinnamoneum* KKP 1658 strain came from the pure culture collection of the Department of Microbiology and Food Biotechnology at the Warsaw University of Life Sciences. The tested strain was stored at −80 °C. The cells were suspended in TSB (tryptone soy broth) with 20% glycerol.

### Preparation of medium and yeast cultures in a bioreactor

The biomass of the *Streptoverticillium cinnamoneum* KKP 1658 strain was obtained by inoculating the microorganism into 100 ml of TSB medium (casein peptone 17 g/L, soy peptone 3 g/L, NaCl 5 g/L, K_2_HPO_4_ 2.5 g/L, glucose 2.5 g/L) with a pH of 7 and incubated for 72 h at 28 °C and 160 rpm on an incubator shaker (Eppendorf Innova 44 Incubator Shaker). Then, in order to continue the growth, 30 mL of the previously prepared culture was added to 270 mL of fresh TSB medium in three repetitions.

A total of 2700 mL of MTG test substrate was prepared, containing ingredients such as soluble starch (20 g/L), the peptone-based nutrient Aminobak (10 g/L), corn steep liquor (10 g/L), yeast extract (2 g/L) and mineral salts (KH_2_PO_4_ – 2 g/L, Na_2_HPO_4_∙12H_2_O – 2 g/L, MgSO_4_∙7H_2_O – 1 g/L), in variants with pH 5.5, 6.0, and 6.5 and nitrogen source content of 1%, 2%, and 4% (as the sum of corn steep liquor and Aminobak in a 1:1 ratio). The medium was sterilised in an autoclave (121 °C, 15 min; Hirayama, Japan) and then inoculated into the bioreactor with a 24-h inoculum. The cultures were maintained at 28 °C with stirring at 200 rpm and aeration using compressed air at a flow rate of 2.0 vvm. Samples for the transglutaminase activity analysis were taken after 24, 48, and 72 h of cultivation.

### Biomass yield determination

The biomass yield of *S. cinnamoneum* KKP 1658 was determined after centrifuging (Eppendorf 5810 Centrifuge, Germany) (4500 rpm; 10 min) 30 mL of culture liquid in dry, previously weighed falcons. The supernatant was separated to determine the transglutaminase activity, and the sediment was dried (SML Zalmed dryer, Poland) at 80 °C until a constant weight was obtained. The biomass yield after centrifugation and drying in three repetitions was calculated per 1 L of substrate and expressed in grams of dry matter (g _d.m__._/L).

### Transglutaminase activity determination

The enzymatic activity of transglutaminase was determined using the commercial Microbial Transglutaminase Assay Kit Art. No. Z009 (Zedira GmbH, Darmstadt, Germany). The kit uses the substrate Z-Gln-Gly (N2-[(phenylmethoxy)carbonyl]-L-glutaminyl-glycine, C_15_H_19_N_3_O_6_) as an amine acceptor and hydroxylamine as an amine donor. In the presence of MTG, the hydroxylamine reacts with the Z-Gln-Gly substrate to form Z-glutamyl-hydroxamic acid glycine, which forms a coloured complex with iron (III), detected spectrophotometrically (BIO-RAD SmartSpec 3000 spectrophotometer, Poland) at a wavelength of 525 nm.

### Protein precipitation

Ammonium sulphate (Chempur, Poland) was used to precipitate proteins from the culture fluid. A sample of the culture fluid was placed on a magnetic stirrer under refrigeration, keeping it on ice. Then, a saturated solution of ammonium sulphate (NH_4_)_2_SO_4_ with a concentration of 750 g/L was added in such a way as to obtain a final salt concentration of 10% in the sample. The sample was incubated in ice for about 2 h, which allowed for adequate protein precipitation.

After incubation, the sample was centrifuged for 15 min at 10,000 rpm (Eppendorf, Germany), and the supernatant was removed. The pellet was washed with cold 90% acetone, centrifuged again, the liquid was removed, and the pellet was dried. Then, the sediment was suspended in a sterile 50 mM citrate-phosphate buffer with a pH of 5.6, using a volume equal to that which was taken at the beginning (Duong-Ly and Gabelli 2014).

### Determination of protein content

The Lowry method et al. (1951) was used to determine the concentration of enzymatic proteins in protein sludge and supernatant. It is based on the presence of peptide bonds, cysteine, and aromatic amino acids (tyrosine and tryptophan) in proteins.

The following was used to prepare the copper reagent: solution A: 2% sodium carbonate solution in 0.1 M NaOH, solution B: 2% aqueous solution of sodium potassium tartrate, solution C: 1% aqueous solution of copper (VI) sulphate pentahydrate.

The solutions were mixed in a ratio of 98 mL A/1 mL B/1 mL C. Then, 1 mL of the test sample or water (control sample) was mixed with 5 mL of the copper reagent. After 10 min, 0.5 mL of Folin-Ciocalteu reagent was added. The samples were incubated for 30 min at room temperature, after which the absorbance was measured at 750 nm in relation to the blank sample. The measurement was carried out three times for each sample.

Standard albumin solutions with concentrations ranging from 100 to 1000 μg/mL were prepared. The absorbance was measured at a wavelength of 750 nm, with three parallel measurements for each concentration. Based on the averaged results, a regression equation was calculated, showing the dependence of absorbance on protein concentration.

### Concentration and purification of transglutaminase

After centrifuging the culture fluid from the bioreactor culture, the obtained supernatant was partially purified by ultrafiltration. A Sartorius Vivaflow 200 system (Sartorius AG, Germany) with a PES (polyethersulfone) membrane with a molecular weight cut-off (MWCO) of 100 kDa and a filtration area of 200 cm^2^ was used. The process was carried out according to the manufacturer’s instructions at a temperature of approximately 4 °C and an operating pressure of up to 3 bar. Filtration was carried out until approximately 1/10 of the culture volume remained.

### Determination of the enzyme’s molecular weight

The molecular weight of the enzyme was estimated using the SDS-PAGE method. The electrophoresis was carried out on a polyacrylamide gel with a composition of 10% (separating gel) and 3% (thickening gel). Protein samples with a volume of 25–50 µL were mixed in a 2:1 ratio with reducing buffer and heated to 100 °C for 5 min (Eppendorf, Thermomixer Comfort). Electrophoresis was carried out at a constant current of 40 mA for 60 min (Bio-Rad PowerPac Universal), and the proteins were visualised by staining with Coomassie Blue R-250, compared with reference proteins of known molecular weights (Broad Range Bio-Rad).

### Determination of thermostability and pH stability of the enzyme

To evaluate the thermostability of microbial transglutaminase, the enzyme was incubated for 2 h at temperatures ranging from 20 to 55 °C in 50 mM phosphate buffer (pH 7.0). After incubation, the residual activity was measured under standard assay conditions. Relative activity (%) was calculated based on the highest observed average activity.

pH stability was assessed in a similar manner: the enzyme was incubated for 2 h at 28 °C in buffers with a pH range of 4.0–9.0, followed by activity measurement. The results were expressed as residual activity (%) relative to the maximum.

### Determination of time-dependent enzyme stability

The time-dependent stability of the microbial transglutaminase was tested by incubating it at 28 °C (Binder, Germany) for various lengths of time, from 30 to 300 min, in 50 mM phosphate buffer at pH 7.0. The results were expressed as residual activity (%) relative to the maximum.

### Analytical methods

The Box-Behnken experimental design was used to optimise the parameters affecting the production of microbial transglutaminase, which takes into account three levels of three different variables. The process of statistical analysis, experiment design, and model creation was carried out using Statistica software version 13.1. The significance of differences between the mean values in individual groups was verified using the Tukey HSD test at the significance level *α* = 0.05.

## Results

Optimising biotechnological processes is key to increasing the production efficiency of enzymes such as microbial transglutaminase. Precisely adjusting the parameters of microorganism cultivation makes it possible to achieve higher enzyme activity and improve the quality of the final product (Marin-Sanguino et al. 2007; Kolotylo et al. 2024b). Current research is focussing on optimising MTG production using non-genetically modified strains.

### Optimisation of conditions for the microbial production of transglutaminase on a bioreactor scale

Initial research on optimising MTG production by the *Streptoverticillium cinnamoneum* KKP 1658 strain was carried out on a laboratory scale, using a working volume of 100 mL of substrate (Kolotylo et al. 2024a, 2024b). This article focuses on transferring the process of producing microbial transglutaminase to a bioreactor scale with a working volume of 3 L, while maintaining the same process parameters optimised on a laboratory scale.

Table 1 shows selected ranges of nitrogen doses (A), culture duration (B), and initial pH (C), which have been coded as −1, 0, and 1, where −1 corresponds to the minimum value of the range and 1 to the maximum value of the range used in the present work.

**Table 1:** Values of coded levels used for the experimental design

**Factors** (independent variables)	**Symbols**	**Actual levels of coded factors**		
		**−1**	**0**	**1**
Dose of nitrogen (%)	A	1	2	4
Time (h)	B	24	48	72
Initial pH	C	5.5	6	6.5

**Fig. 1:**
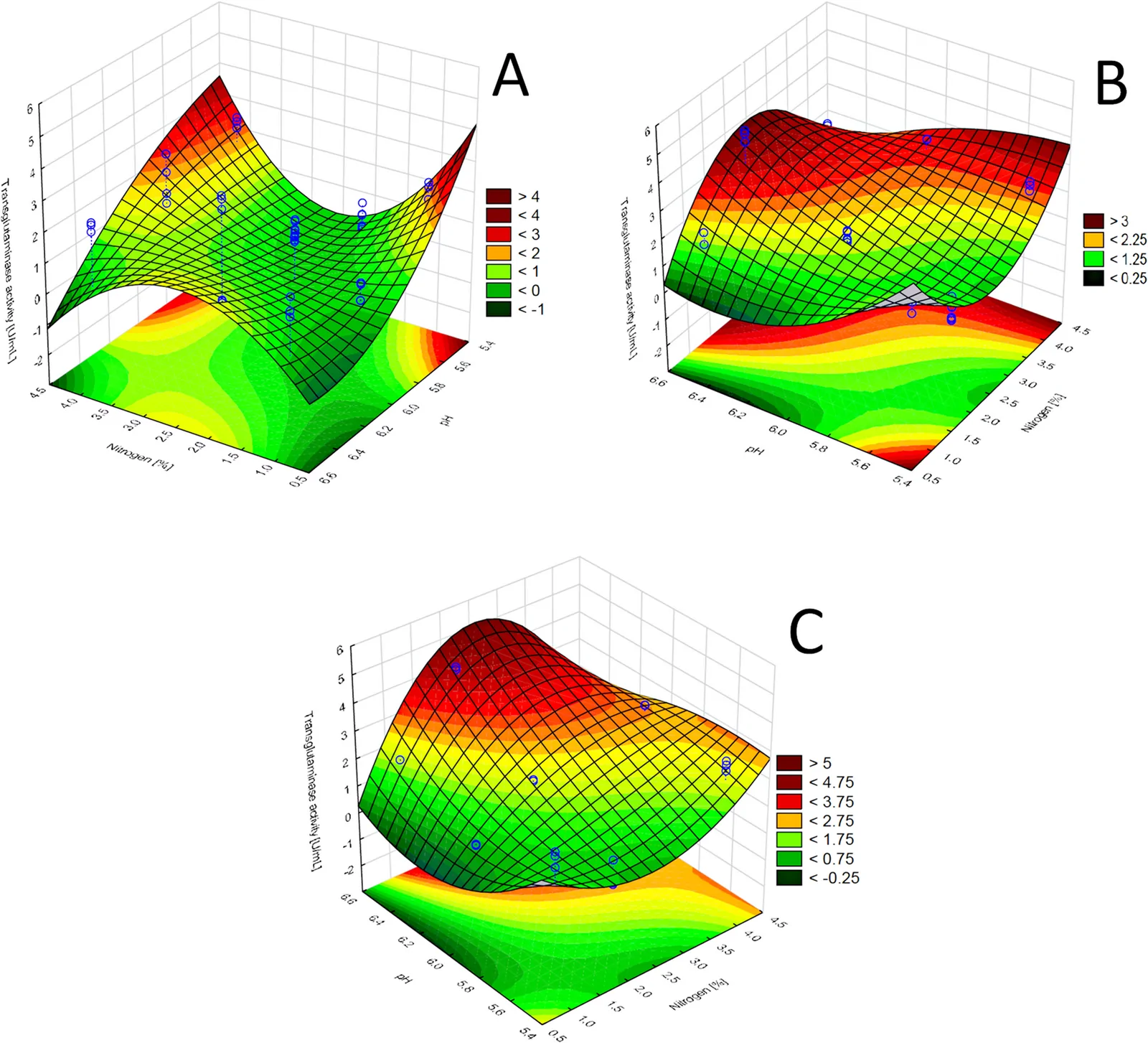
Response surface for MTG production by *Streptoverticillium cinnamoneum* KKP 1658 after. **A** 24 h of culture, **B** 48 h of culture, and **C** 72 h of culture. The interaction between dose of nitrogen and initial pH

To optimise the conditions for production of transglutaminase by the *Streptoverticillium cinnamoneum* KKP 1658 strain, an experiment based on a fractional three-factor design according to Box-Behnken methodology was carried out, taking into account three experimental factors (Table 1). The study involved 15 bioreactor cultures, comprising 12 different combinations of variables and 3 replicates at the central point. The choice of three variables—nitrogen source dose, initial pH, and culture time—was based on previous preliminary studies conducted in shake flasks (Kolotylo et al. 2024a, 2024b), which showed their significant influence on microbial transglutaminase production by *Streptoverticillium cinnamoneum* KKP 1658. Based on the experimental data obtained, coefficients were determined for the quadratic model, and response surface plots were used to visualise the influence of individual factors (Fig. 1). The results of the experiment according to the Box-Behnken design are summarised in Table 2.

**Table 2:** Box-Behnken design with actual and coded levels of variables and actual values of responses

Run number	Nitrogen (%)	pH	Time (h)	Transglutaminase activity (U/mL)
1	1	5.5	48	**2.096** ± 0.29
2	1	6.5	48	**1.518** ± 0.32
3	4	5.5	48	**2.568** ± 0.18
4	4	6.5	48	**2.179** ± 0.16
5	2	5.5	24	**0.236** ± 0.06
6	2	6.5	24	**1.081** ± 0.05
7	2	5.5	72	**0.800** ± 0.23
8	2	6.5	72	**4.185** ± 0.22
9	1	6	24	**0.090** ± 0.01
10	4	6	24	**1.371** ± 0.33
11	1	6	72	**0.643** ± 0.03
12	4	6	72	**2.786** ± 0.34
13	2	6	48	**1.524** ± 0.11
14	2	6	48	**1.518** ± 0.19
15	2	6	48	**1.940** ± 0.17

The regression coefficients were calculated and the fitted equations for predicting transglutaminase activity (*X*) are given below:

1 $$\begin{aligned}X\:&=\:230.26\;-\;96.66\:\times\:A\:+\:8.31\:\times\:A^2\;-\;68.48\:\times\:C\:+\:5\:\times\:C^2\:+\:24.98\:\times\:A\:\times\:C\;-\;1.53\:\times\:A\:\times\:C^2\;\\&-\;1.32\:\times\:A^2\:\times\:C\:+\:0.06\:\times\:B\:\times\:A\;-\;0.01\:\times\:B\:\times\:A^2\:+\:0.05\:\times\:B\:\times\:C\;-\;14.67\;\end{aligned}$$

where *A* is the nitrogen dose, *B* is the cultivation time, and *C* is the initial pH of the cultivation.

**Fig. 2:**
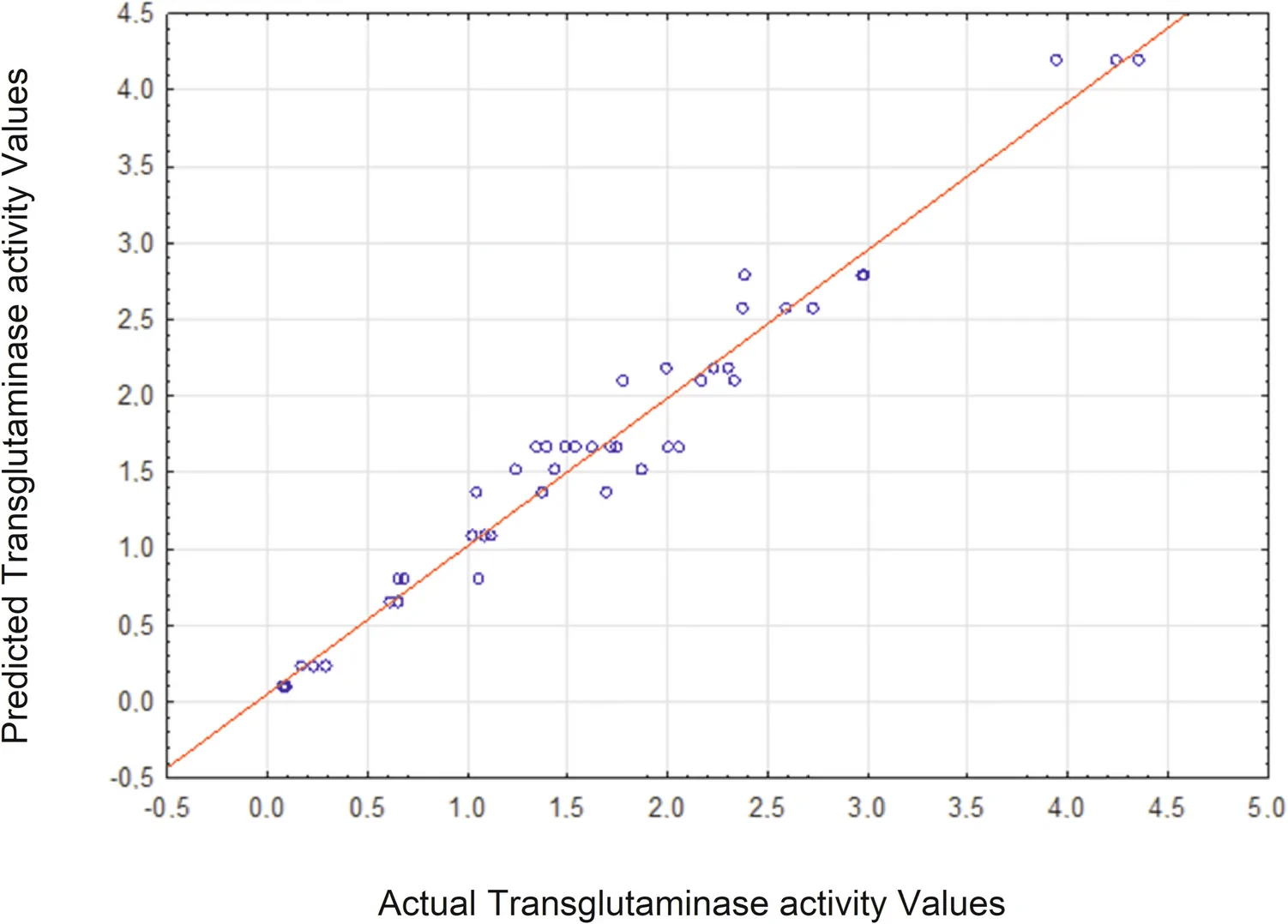
Predicted vs. actual values for transglutaminase activity

The predicted values, determined on the basis of the above equation, showed very good agreement with the experimental results (Fig. 2). This confirms the usefulness of the quadratic model used in the current experimental conditions.

The suitability of the experimental model for the results of transglutaminase activity was evaluated based on the coefficient of determination *R*^2^. The *R*^2^ value of 95.34% (Table 3) indicates that the model is able to explain more than 95% of the total variability of the results. According to Jiang (2010), regression models with a coefficient of determination exceeding 0.9 (90%) are characterised by a very high correlation. To assess the significance of the factors in the model, a significance level of *α* = 0.05 was used; *p*-values exceeding this threshold were considered non-significant, while values below it indicated significance (Table 3).

**Table 3:** Analysis of variance for response surface quadratic model obtained from experimental results

Source	Sum of squares	df	Mean square	*F*-value	*p*-value
A	4.85121	2	2.42560	47.2529	< 0.0001
B	1.73756	2	0.86878	16.9246	< 0.0001
C	12.59189	2	6.29595	122.6508	< 0.0001
AB	12.12454	3	4.04151	78.7324	< 0.0001
AC	1.64139	2	0.82069	15.9878	< 0.0001
BC	4.83664	1	4.83664	94.222	< 0.0001
Pure total	1.64263	32	0.05133	-	-
Core total	48.51435	44	-	-	-
*R*^2^	0.9534	-	-	-	-

**Fig. 3:**
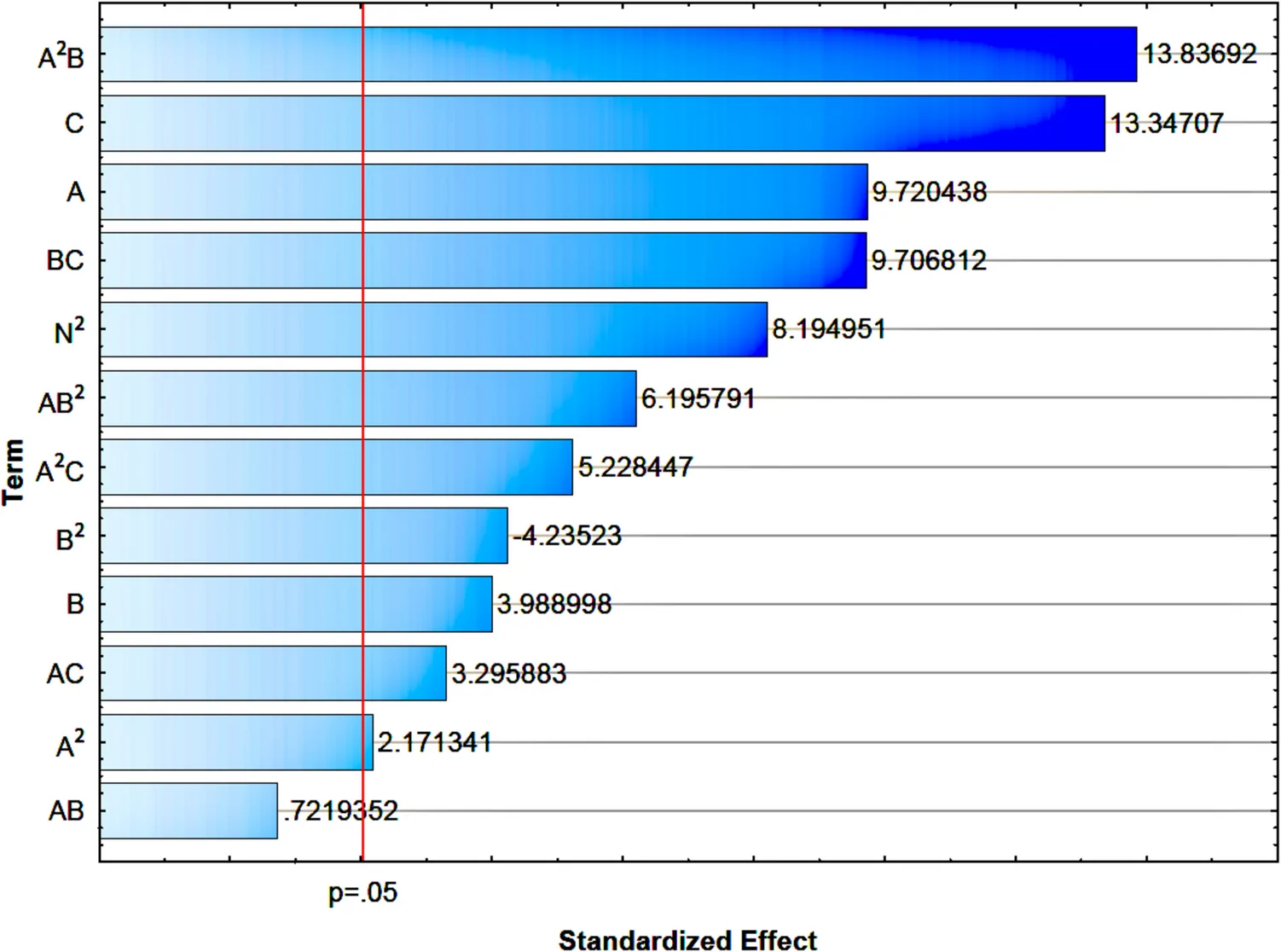
Pareto chart of standardised effect (response is transglutaminase activity (U/mL)). **A** The nitrogen dose, **B** the cultivation time, and (**C**) the initial pH of the cultivation

The influence of individual components of Eq. (1) and their mutual interactions (linear and quadratic) was illustrated using a Pareto chart, taking into account the standardised values of the Student’s *t*-test and a significance threshold of 0.05 (Fig. 3).

Pareto chart analysis indicates that the quadratic interaction between nitrogen dose and culture time (*A*^2^*B*) exerted the key effect on microbial transglutaminase (MTG) activity, which suggests the importance of non-linear relationships in process optimisation. This result emphasises that both the level of the nitrogen dose and the incubation time significantly determine the efficiency of enzyme production, and their appropriate adjustment is crucial for obtaining high enzymatic performance. The quadratic factor indicates the most significant impact of the middle level of the nitrogen source dose (2%). In contrast, the linear factor B indicates that the impact of this factor on transglutaminase production increases with the extension of the culture time (72 h). Another critical factor is the pH (C), which showed a powerful linear impact on MTG activity. A high initial pH level (6.5) significantly affects MTG production among the tested acidity ranges. This confirms previous studies, which indicated the necessity of precise pH level adjustment to ensure optimal conditions for enzymatic reactions.

A significant effect was also recorded for the linear factor of the nitrogen dose (A) and the interaction between pH and culture time (BC), indicating these parameters’ mutual interaction in the fermentation process. It is worth emphasising that these interactions, although less significant than *A*^2^*B* and *C*, still have a noticeable effect on enzyme activity and should not be ignored in further optimisation steps.

Less significant factors, such as culture time (B) and the interaction of nitrogen dose with time (AB), had a limited impact on process efficiency, which suggests that their precise regulation may not be critical. Nevertheless, it should be noted that the low significance of these variables may be the result of specific experimental conditions and should be verified in further studies.

In conclusion, optimisation of the MTG production process should focus primarily on fine-tuning the pH level and understanding and controlling the non-linear interactions between nitrogen dose and cultivation time. Focusing on these key variables can significantly increase enzymatic efficiency while reducing the need for excessive control of less influential factors. These results provide valuable guidance for further improving the fermentation process on a larger industrial scale.

### Analysis of the influence of breeding parameters on enzyme activity

A–C show three-dimensional response surface plots developed from the predictive quadratic model. In the surface plots, the interaction between pH and nitrogen concentration was analysed at three fixed levels of cultivation time: 24 h (Fig. 1 A), 48 h (Fig. 1B), and 72 h (Fig. 1 C). This approach allows for the evaluation of two-factor interactions while keeping the third factor constant, in accordance with multifactorial experimental design principles. The graph showing the surface area after 24 h of bioreactor cultivation (Fig. 1 A) shows that the highest enzymatic activity is achieved at a low pH (approximately 5.5–6) and higher nitrogen doses in the 3–4% range. In this phase of enzyme cultivation, lower pH values seem to favour more intensive transglutaminase activity (approximately 4 U/mL), which suggests the necessity of precise pH control in the early stages of fermentation.

The graph of the response surface area after 48 h of cultivation (Fig. 1B) indicates that the optimal conditions for maximum enzyme activity (approximately 4 U/mL) shift towards a higher pH (approximately 6.0–6.5) while maintaining relatively high nitrogen doses (2–4%). Areas of low enzyme activity (< 1.25 U/mL) are visible at low pH and low nitrogen concentration, emphasising these parameters’ key role in the later phases of cultivation. In this stage, enzyme activity increases as environmental conditions such as pH and nutrient availability stabilise.

The response surface plot after 72 h of culture (Fig. 1C) shows the highest transglutaminase activity (approximately 5 U/mL), achieved at a pH of roughly 6.5 and nitrogen doses close to 3%. During this time, the enzyme reaches its maximum activity, which is consistent with the trends observed in the previous stages. It is also worth noting that low pH (below 6.0) and lower nitrogen doses (below 2%) result in a marked decrease in enzyme activity, which may indicate a limited ability of cells to produce the enzyme effectively under such conditions.

To summarise, the results indicate that the key parameters affecting transglutaminase activity are pH and nitrogen dosage, and their significance varies depending on the culture time. The analysis shows that the optimal conditions for enzyme production include a pH of approximately 6.5, a nitrogen dose of 2.5–3% and 72 h of cultivation.. This combination of parameters allows for maximum enzyme activity, as confirmed by the experimental data obtained.

### Verification of optimal culture conditions in the bioreactor

Taking the results obtained into account, the optimal cultivation conditions were defined as a pH of 6.5, a nitrogen dose of 2.75% (a combination of Aminobak and corn steep liquor in a 1:1 ratio, according to our preliminary studies Kolotylo et al. 2024a, 2024b), and a 72-h cultivation time. These conditions were selected based on the analysis of the response surface plots, which allowed us to identify a stable operating range rather than relying solely on the mathematical output of the model. The next stage was to carry out the cultivation using the selected optimal parameters and obtain the enzyme, which, after precipitation from the culture fluid, was subjected to further research (molecular weight, thermostability, and pH stability) (Table 4). The process of precipitating proteins from a solution is based on the use of a precipitating agent that destabilises the protein molecules. Adding this agent removes the stabilising shell of water dipoles, which exposes hydrophobic regions in the protein molecules. As a result, they become less soluble, which promotes their aggregation and precipitation from the solution (Goldring 2019).

**Table 4:** Results of post-culture fluid analysis

Factors	Activity MTG (U/mL)	Protein content (mg/mL)	Specific enzyme activity (U/mg)	Purification fold	Recovery* (%)
Culture fluid (72 h)	4.29 ± 0.08	6.17 ± 0.61	0.70	1.00	100
Fluid after ultrafiltration	10.87 ± 0.15	9.09 ± 0.71	1.20	1.71	253
Proteins precipitated with ammonium sulphate	22.0 ± 0.05	13.40 ± 0.31	1.64	2.34	513

^*^Due to concentration effects during ultrafiltration and precipitation, the activity values are expressed per 1 mL of sample. The actual recovery rate may vary depending on the final volume of the fractions

The analysis of the culture fluid showed that after 72 h of incubation, the biomass yield of *S. cinnamoneum* KKP 1648 was 14.62 ± 0.56 g/L, and transglutaminase activity after centrifugation was 4.29 U/mL, with a protein content of 6.17 mg/mL. The predicted transglutaminase activity, determined based on a response surface modelling (RSM) method, was 4.47, with a 95% confidence interval ranging from 4.18 to 4.76 (Table 5). The experimentally observed value of 4.29 fell within this range, indicating satisfactory agreement between the model predictions and the experimental results. These results confirm the reliability of the developed model for estimating transglutaminase activity under the tested conditions.

**Table 5:** Validation of the RSM model for transglutaminase activity estimation

Parameter	Predicted value	Observed value	95% Confidence interval
Activity MTG [U/mL]	4.47	4.29	4.18–4.76

After the ultrafiltration process (Sartorius Vivaflow 200, MWCO 100 kDa), the enzyme activity increased to 10.87 U/mL, and the specific activity increased from 0.7 to 1.2 U/mg. The highest parameter values were achieved after precipitation of proteins with ammonium sulphate. After this process, the MTG activity was 22 U/mL, which translated into the highest specific activity of 1.64 U/mg.

The use of ultrafiltration and protein desalting processes improved the activity and purity of the obtained microbial transglutaminase, which was an essential step for further analyses of the enzyme preparation.

### Properties of the isolated transglutaminase

**Fig. 4:**
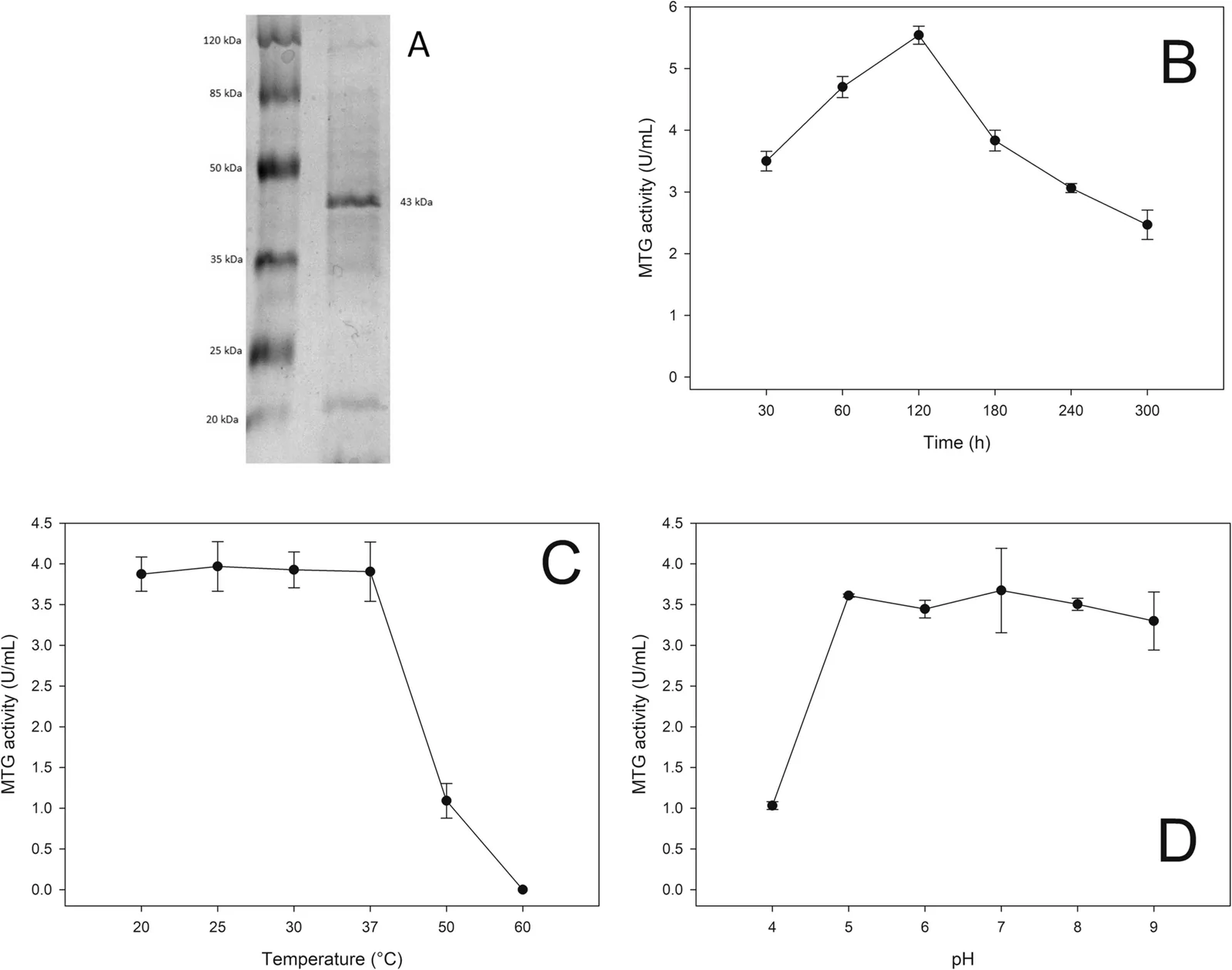
Biochemical characteristics of transglutaminase. **A** SDS-PAGE of purified transglutaminase from *Streptoverticillium cinnamoneum* KKP 1658, **B** effect of time-dependent stability on the transglutaminase activity, **C** results of determination of the thermostability of transglutaminase, and (**D**) pH stability on the activity of transglutaminase

The isolated transglutaminase showed a clear single protein band in the SDS-PAGE analysis (Fig. 4A, File 1S). The molecular weight of the protein was estimated at 43 kDa based on its electrophoretic mobility compared to the standard proteins used. Although ultrafiltration significantly increased MTG activity, SDS-PAGE analysis revealed the presence of small bands below 20 kDa, suggesting the presence of residual contaminants. This may affect the accuracy of the specific activity estimate, and further purification using lower MWCO membranes is recommended to improve the purity of the resulting enzyme.

For the next part of the study, the obtained transglutaminase (stored at −80 °C) with an activity of 22 U/mL was thawed and diluted with a citrate-phosphate buffer to obtain a larger volume of enzyme for the experiments. After dilution with buffer, a transglutaminase solution with an activity of 3.20 ± 0.23 U/mL was obtained. The time-dependent stability of the tested transglutaminase was evaluated by incubation at 28 °C for 300 min (Fig. 4B). The enzyme activity reached its maximum value after 120 min of incubation (5.54 U/mL), while significant decreases in activity were observed at the end of the experiment—after 240 min. The obtained results indicate high stability of the obtained enzyme from the tested strain *S. cinnamoneum* KKP 1648.

The thermostability of enzyme activity was then determined after incubating the transglutaminase solution for 2 h at temperatures between 20 and 60 °C (Fig. 4C). The tested enzyme showed a wide range of thermostability, as a similar average enzyme activity (3.87–3.97 U/mL) was obtained in the range of 20–37 °C. A significant reduction in the activity of the tested transglutaminase occurred at 50 °C, with a decrease in activity by 75% (1.09 U/mL) compared to the results for the temperature range of 20–37 °C. The temperature of 60 °C completely deactivated the tested enzyme. Interestingly, MTG activity increased during the first 120 min of incubation, which may indicate a transient effect of thermal activation. This behaviour is atypical for bacterial transglutaminases but was previously observed in the studies of De Souza et al. (2009). Authors showed that transglutaminase isolated from *Bacillus circulans* shows increased enzymatic activity after short-term heating at 40–45 °C before its thermal inactivation begins. This is explained by possible conformational changes of the enzyme leading to better access to the active site. Similar behaviour was observed in the present study, which may indicate a similar activation mechanism.

To evaluate the pH stability of transglutaminase, the enzyme was pre-incubated at 28 °C for 2 h in a citrate-phosphate buffer with a pH ranging from 4.0 to 9.0 (Fig. 4D). Transglutaminase was characterised by high residual activity in a wide pH range of 5.0–8.0 (3.45–3.67 U/mL). An acidic environment (below pH 5.0) drastically reduced the enzyme activity to 1.03 U/mL (70% of the activity at pH 5.0), compared to a basic pH, where a mild decrease in activity (3.3 U/mL) was observed in a buffer at pH 9.0. The highest residual activity was observed after incubation in buffer at a pH of 7.0, where it reached an average value of 3.67 U/mL.

## Discussion

Industrial-scale MTG production is mainly carried out in bioreactors, which provide precise control over fermentation conditions. Bioreactors allow constant culture parameters to be maintained, which is crucial for obtaining a high enzyme yield. In this study, optimisation of the bioreactor culture conditions of *Streptoverticillium cinnamoneum* KKP 1658 was carried out in order to increase the production of transglutaminase. The maximum transglutaminase activity (4.29 U/mL) was obtained at pH 6.5, a nitrogen dose of 2.75%, a culture time of 72 h, and a stirring speed of 200 rpm. In the study by Zilda et al. (2017), the optimal MTG production in the bioreactor for the *Streptomyces thioluteus* TTA 02 SDS 14 strain was achieved at a stirring speed of 150 rpm and an incubation time of 48 h. The optimal production conditions for MTG for *S. thioluteus* TTA 02 SDS 14 are pH 6.0 and 30 °C. It is worth noting that after the optimisation process, the researchers obtained a maximum transglutaminase activity of 0.135 U/mL.

Although the stirring speed was not included in the optimisation process for *S. cinnamoneum* KKP 1648, it plays an essential role in producing enzymes, including microbial transglutaminase. Mixing influences the oxygenation level of the culture, which is a critical factor in the growth of microorganisms and the biosynthesis of enzymes. As demonstrated in the study by Aidaroos et al. (2011), *Streptomyces hygroscopicus* WSH03-01 required intensive mixing at 500 rpm in a bioreactor to achieve optimal transglutaminase production after 72 h of incubation (71.75% increase in activity compared to the results before optimisation). *Streptomyces mobaraensis* achieved the highest enzymatic activity (3.2 U/mL) at a stirring speed of 200 rpm (Guerra-Rodríguez and Vázquez 2014). Lower stirring speeds can lead to an extended enzyme production time, which was observed at 100 rpm, when a longer incubation period was required to reach maximum transglutaminase activity in the study by Zilda et al. (2017). In the present research, although the stirring speed was not a variable in the optimisation model, the highest transglutaminase activity of approximately 5.0 U/mL was achieved after 72 h of cultivation at pH 6.5 and a nitrogen dose of 2.75%, as shown in the response surface plots. This value significantly exceeds the maximum activities reported for *S. mobaraensis* or *S. hygroscopicus*, indicating a high efficiency of enzyme biosynthesis in *S. cinnamoneum* KKP 1658 under the applied conditions. This suggests that carefully adjusted medium composition and pH can partially compensate for the lack of aeration optimisation. Nevertheless, future work should consider including stirring speed in the design to verify its potential impact for *S. cinnamoneum* KKP 1648.

In a study on microbial transglutaminase, 200 bacterial strains were isolated from soil and wastewater samples, five of which showed the ability to synthesise the enzyme (Sorde and Ananthanarayan 2019). The highest activity was obtained for the strain *Bacillus nakamurai* NRRL B 41091 (B4), with 3.95 ± 0.04 U/mL, and *Bacillus subtilis* BCRC 10255 (C2), with 2.65 ± 0.17 U/mL, after optimisation of fermentation conditions. This represents an increase from the initial activities of 1.71 ± 0.2 U/mL and 1.61 ± 0.17 U/mL, for isolates B4 and C2, respectively. The maximum enzyme activity of strains B4 and C2 was obtained in a medium containing peptone (20 g/L), starch (20 g/L), yeast extract (2 g/L) and mineral salts (Mg_2_SO_4_·7H_2_O – 2 g/L, K_2_HPO_4_ – 2 g/L, KH_2_PO_4_ – 2 g/L). The optimal culture parameters included a pH of 6.5–7.5 for isolate B4 and 7.5 for C2, a temperature of 35 °C, and a stirring speed of 180 rpm. Maximum enzyme activity was achieved in the stationary growth phase, between 48 and 60 h of cultivation. The results confirm that environmental conditions are crucial for the effective production of MTG, and that appropriate optimisation can significantly increase the enzyme’s efficiency. The cultivation of *S. cinnamoneum* KKP 1658 under similar pH (6.5) and nitrogen conditions resulted in a maximum enzyme activity of 5.0 U/mL after 72 h, as determined from the response surface plots. This activity surpasses the values reported for both *Bacillus* strains, indicating a higher production potential under the applied medium composition. Furthermore, thermostability and pH stability tests demonstrated that the enzyme retained high activity under a range of conditions. Maximum residual activity after 2-h incubation was observed at 25–30 °C (relative activity > 95%). pH stability analysis showed that the enzyme maintained > 90% relative activity in the range of pH 5.0–8.0, with a peak activity at pH 7.0 (3.67 U/mL). In time-dependent stability assays, transglutaminase activity increased within the first 2 h of incubation, reaching 5.54 U/mL, confirming the high functional stability of the enzyme and its suitability for extended processing conditions.

The pH stability range is a key factor influencing the microbiological activity of transglutaminase and its use in various biotechnological processes. As demonstrated by Macedo et al. (2011), transglutaminase produced by the strain *Streptomyces* sp. CBMAI 837 exhibits the highest activity in the pH range of 6.0–6.5, which is consistent with the results obtained in this study. However, it is worth noting that some strains may have different environmental requirements. For example, *Bacillus subtilis* produces transglutaminase with maximum activity at pH 8.2 (Suzuki et al. 2000). These differences are most likely due to the structural specificity of the enzyme and the optimal conditions for the growth of individual microorganisms. The results obtained in our study, indicating an optimal pH of 6.5 for *Streptoverticillium cinnamoneum* KKP 1658, are consistent with previous reports on microbiological sources of transglutaminase. This emphasises the importance of precisely controlling this parameter in fermentation to achieve maximum enzyme activity.

The study by Sorde and Ananthanarayan (2019) showed that the optimal pH for the activity of transglutaminase produced by the two isolates, *Bacillus nakamurai* NRRL B 41091 (B4) and *Bacillus subtilis* BCRC 10255 (C2), was 6.0. The enzyme was stable over a wide pH range (5.0–9.0); however, the activity of MTG of strain B4 decreased rapidly in acidic conditions, while it was more stable in alkaline environments, which was the opposite of the observations for the isolate C2. The optimal pH for the activity of MTG for the *Streptomyces hygroscopicus* strain WSH03-13 was found to be in the range of 6.0–7.0, and this enzyme showed pH stability in the range of 5.0–8.0 (Cui et al. 2007).

The transglutaminase isolated in our study from *S. cinnamoneum* KKP 1648 was characterised by a pH stability similar to *B. nakamurai* NRRL B 41091 and *S. hygroscopicus* WSH03-13. MTG stability was observed in the pH range of 5.0–9.0, while an acidic environment (pH < 5.0) rapidly reduced the determined activity. The stability of transglutaminase strains B4 and C2 was maintained in the temperature range of 0–50 °C, after which there was a decrease in activity. In a study by Zilda et al. (2017), the transglutaminase obtained in a 2 L working volume bioreactor from the *Streptomyces thioluteus* TTA 02 SDS 14 strain was most active at 45–50 °C and at a pH of 6.0. In another study by Wan et al. (2017), the transglutaminase of the *Streptomyces hygroscopicus* H197 strain showed optimal activity at a pH of 6.0–8.0 and was most stable at 40 °C. The transglutaminase obtained from *Streptomyces* sp. CBMAI 837 showed optimal activity at 35–40 °C, and the enzyme was stable over a wide pH range (4.5–8.0) and up to 45 °C. In our study, MTG activity was maintained in the temperature range up to 40 °C, after which deactivation of the enzyme was observed (Fig. 4C). Cui et al. (2007) obtained similar results for the transglutaminase isolated from the soil strain *Streptomyces hygroscopicus* WSH03-13. The enzyme showed optimal activity at a temperature of 37–45 °C, and an increase in temperature above 50 °C completely deactivated the enzyme. These results emphasise the differences in the characteristics of enzymes produced by individual strains and their potential application in various industrial conditions. It is worth emphasising that the level of enzymatic activity of about 17% and 42% of MTG was maintained at 70 °C for isolates B4 and C2, respectively, while the transglutaminase obtained in our research, originating from *S. cinnamoneum* KKP 1648, was found to be inactive at a temperature of 60 °C, which indicates higher thermostability of MTG produced by *Bacillus* bacteria.

In the present study, the molecular weight of the labelled transglutaminase was 43 kDa (Fig. 4A), which is consistent with the values reported for other microbiological transglutaminases. Data presented by Macedo et al. (2011) showed that the molecular weight of the enzyme was approximately 45 kDa, which is similar to the result obtained in this study. Jin et al. (2016) determined that the molecular weight of MTG-TX transglutaminase obtained from *Streptomyces mobaraensis* TX was 37.82 kDa based on the results of liquid chromatography and mass spectrometry. Zhang et al. (2012) determined the molecular weight of MTG isolated from *Streptomyces mobaraensis* DSM 40587 as 38 kDa using SDS-PAGE. Similar values were also recorded by Cui et al. (2007) and Yu et al. (2008), who determined the MTG mass to be 38 kDa. However, it is worth noting that molecular weight differences can result from interspecies differences, purification methods, and electrophoresis conditions. On the other hand, Wan et al. (2017) described an MTG mutant with an increased molecular weight of 67 kDa, which may suggest differences in the protein structure resulting from genetic modifications. The results confirmed that the molecular weight of transglutaminase may vary depending on the microbiological source and the method of its isolation and purification.

Many studies on microbiological transglutaminase have been published, but there is no established specific methodology for purifying this enzyme. According to Zhang et al. (2012), after separating the culture from the microbiological substrate by centrifugation or filtration, the supernatant is concentrated by ultrafiltration or salting out with solid ammonium sulphate or ethanol.

In order to increase the transglutaminase activity of *S. cinnamoneum* KKP 1658 and to increase the purity of the obtained enzyme preparation, the centrifuged culture fluid was subjected to ultrafiltration using the Sartorius Vivaflow 200 system with a molecular weight cut-off (MWCO) membrane of 100 kDa, which allowed the elimination of larger peptides and impurities. After this stage, the enzyme specificity was obtained at the level of 1.2 U/mg, which is an increase of 71.4% compared to the culture fluid. Then, to precipitate the transglutaminase, an ammonium sulphate fractionation process was carried out, leading to an increase of 134.3% (1.64 U/mg) compared to the value for the culture fluid (0.7 U/mg).

Ultrafiltration is often used as the first or intermediate step in the purification process for enzymes such as lipase (Ghutake et al. 2025), endo-pectinase (Krstić et al. 2007), phytase (Rodríguez-Fernández et al. 2013), xylose reductase (Krishnan et al. 2022), bromelain (Nor et al. 2017), lactase (Antecka et al. 2019), dextranosaccharase (Flórez Guzman et al. 2018), and protease (Hemici et al. 2017). Ghutake et al. (2025) increased the production yield of raw lipase 2.29-fold by using ultrafiltration with a 5 kDa MWCO membrane for concentration. In the study by Özer et al. (2018), the efficiency of three methods of beta-lactoglobulin (β-Lg) isolation was compared in terms of purity, yield, and preservation of the native protein structure. The use of ultrafiltration with a 30 kDa MWCO membrane provided the highest degree of purification (43.6-fold increase over whey), far exceeding the results of ion exchange chromatography and hydrolysis with pepsin—a 6.6-fold and 1.4-fold increase over whey, respectively. The authors emphasised that ultrafiltration also preserved the native structure of β-Lg proteins and effectively eliminated high-molecular impurities, in this case bovine serum albumin or immunoglobulin. Yongsawatdigul and Piyadhammaviboon (2007) studied sarcoplasmic proteins (SpC) from tilapia (*Oreochromis niloticus*), which contain a significant amount of transglutaminase. In their study, they used ultrafiltration with a 30 kDa MWCO membrane, which resulted in a 3.6-fold increase in the total transglutaminase activity of the SpC protein concentrate. It is worth noting that despite the increase in enzyme activity from 137 to 498.6 U/mL, the specific activity of the enzyme decreased from 16.4 to 14.4 U/mg, which may indicate that ultrafiltration only concentrates the SpC protein without increasing the purity of transglutaminase. Ultrafiltration is widely used for enzyme concentration and is considered a basic purification step (Rodríguez-Fernández et al. 2013). Combining ultrafiltration with other techniques (e.g. chromatography, immobilisation on synthetic resins) often increases the purification yield and the stability of the enzyme (Ghutake et al. 2025). This is confirmed by research on the purification of transglutaminase, in which alcoholic and sulphate protein precipitation, ultrafiltration, and gel chromatography were used, obtaining an enzyme with a specific activity of 107.86 U/mg. In addition, the purified enzyme showed high stability—its activity remained above 90% after 2 h of incubation at 40 °C, and the pH stability range was between 5.0 and 7.0.

Zhang et al. (2012) characterised the transglutaminase produced by *Streptomyces mobaraensis* DSM 40587. The preparation was purified using ultrafiltration (MWCO 30 kDa) combined with gel chromatography (Sephadex G-75) and high-performance chromatography (SP Sepharose). Due to the significant amount of magnesium chloride in the medium, the addition of ammonium sulphate would cause excessive precipitation of this salt. The authors also decided not to precipitate the ethanol enzyme, claiming a possible loss of transglutaminase activity. The specific activity of MTG in the culture fluid was determined to be 2.93 U/mg, which increased to 3.24 U/mg after the ultrafiltration process—an increase of 10.6%. The use of gel chromatography allowed us to obtain a specific activity of MTG of 8.14 U/mg, while high-performance liquid chromatography resulted in 17.2 U/mg, which is an increase of 177.8 and 487.4%, respectively. Purified transglutaminase showed the highest activity at 55 °C and pH 6.0. The enzyme lost its activity at temperatures above 50 °C. It was stable for 12 h at a pH of 5.0 to 10.0 at 4 °C and for 30 min at a pH of 5.0 to 9.0 at 37 °C.

Jin et al. (2016) purified MTG from *S. mobaraensis* TX isolated from soil using ethanol precipitation, justifying their choice with the higher cost-effectiveness of the process compared to ultrafiltration. In the culture fluid, the specific activity of transglutaminase was found to be 1.75 U/mg, and after ethanol precipitation, a result of 7.02 U/mg was obtained (an increase of 301.1%). In the subsequent purification stages, a cation exchange chromatograph (AKTA Purifier) and a hydrophobic chromatograph (phenyl Sepharose column) were used, and the results obtained were 37.9 and 39.2 U/mg, respectively. Purified transglutaminase showed stability in a wide pH range of 5.0–10.0, with an optimal temperature of 45–50 °C.

The above results indicate that the use of multi-stage purification not only supports the concentration of the enzyme, but also allows it to be further processed without loss of activity, which is crucial in the development of stable enzyme preparations. The increase in specific activity obtained in this study after ultrafiltration (by 71.4%) and after precipitation with ammonium sulphate (134.3%) confirms the effectiveness of these enzyme purification methods, as also indicated by the examples of other studies described above. It is worth noting that despite the increase in transglutaminase activity from 4.29 to 22 U/mL, the maximum specific activity determined did not exceed 1.64 U/mg, which is insufficient compared to the results of 39.2 U/mg reported by Jin et al. (2016) and 17.2 U/mg by Zhang et al. (2012). In the future, the purification methods used in this study should be integrated with chromatographic methods to obtain competitive specific activity results.

## Conclusion

The production process in a bioreactor allows for precise scaling of enzyme production and cost reduction, making it the preferred method for mass production. This study determined the optimal conditions for the microbial production of transglutaminase (MTG) by the *Streptoverticillium cinnamoneum* KKP 1648 strain under bioreactor conditions. Based on the results obtained, the optimal culture parameters were determined to be a pH of 6.5, a nitrogen dose of 2.75% (using Aminobak and waste corn steep liquor in a 1:1 ratio), and a culture time of 72 h. The agreement between the predicted and observed values confirms the correctness of the developed model and its usefulness for predicting transglutaminase activity under the tested conditions. After the cultivation, the enzyme was isolated and subjected to further characterisation. In the conducted studies, after the ultrafiltration process, an increase in the specific activity of the enzyme by 71.4% was observed in relation to the cultivation fluid, which confirms this method’s effectiveness in increasing the purity of the protein preparation. Further precipitation with ammonium sulphate resulted in a specific activity of 1.64 U/mg (an increase of 134.3% compared to the culture fluid).

Electrophoretic analysis showed that the tested transglutaminase has a molecular weight of 43 kDa, which is consistent with the values obtained for enzymes from other microbiological sources. In terms of time-dependent stability, the enzyme activity reached a maximum after 120 min of incubation at 28 °C (5.54 U/mL), but a significant decrease in activity was observed after 240 min. The enzyme studied had a wide range of thermostability (20–37 °C), with a significant decrease in activity at 50 °C (75% decrease). Complete inactivation of the enzyme occurred at 60 °C.

In terms of pH stability, the tested transglutaminase showed high activity in a wide pH range (5.0–8.0), reaching a maximum value at pH 7.0 (3.67 U/mL). In an acidic environment (pH < 5.0), a sharp decrease in activity was recorded (1.03 U/mL), while in an alkaline environment (pH 9.0), the decrease in activity was less pronounced (3.3 U/mL).

The results indicate that *S. cinnamoneum* KKP 1648 is an effective MTG producer in bioreactor conditions, and the optimised fermentation process allows obtaining an enzyme with stable properties in a wide range of temperatures and pH. It is worth emphasising that the use of waste corn steep liquor as a component of the culture medium contributed to the optimisation of the process. Future research should focus on the possibility of using even more waste raw materials as components of microbiological media, which would further reduce production costs and the environmental burden. Practical applications of the obtained transglutaminase in food technology should also be explored, especially in meat, bakery, and dairy products, where the enzyme can significantly improve the texture and quality of the final product.

## Supplementary Information

Below is the link to the electronic supplementary material.

ESM 1 Supplementary Material 1: Figure of the whole polyacrylamide gel with transglutaminase enzyme fractions. (PNG 378 KB)

High Resolution Image (TIF 3.96 MB)

## Data Availability

Data sharing is not applicable to this article as no datasets were generated or analysed during the current study.
