# Supplementary figures and images for: Characterisation and optimisation of microbial production of transglutaminase produced by Streptoverticillium cinnamoneum
Source: Appl Microbiol Biotechnol. 2025 Oct 16;109(1):228. doi: 10.1007/s00253-025-13606-y (PMC12532617; doi:10.1007/s00253-025-13606-y)

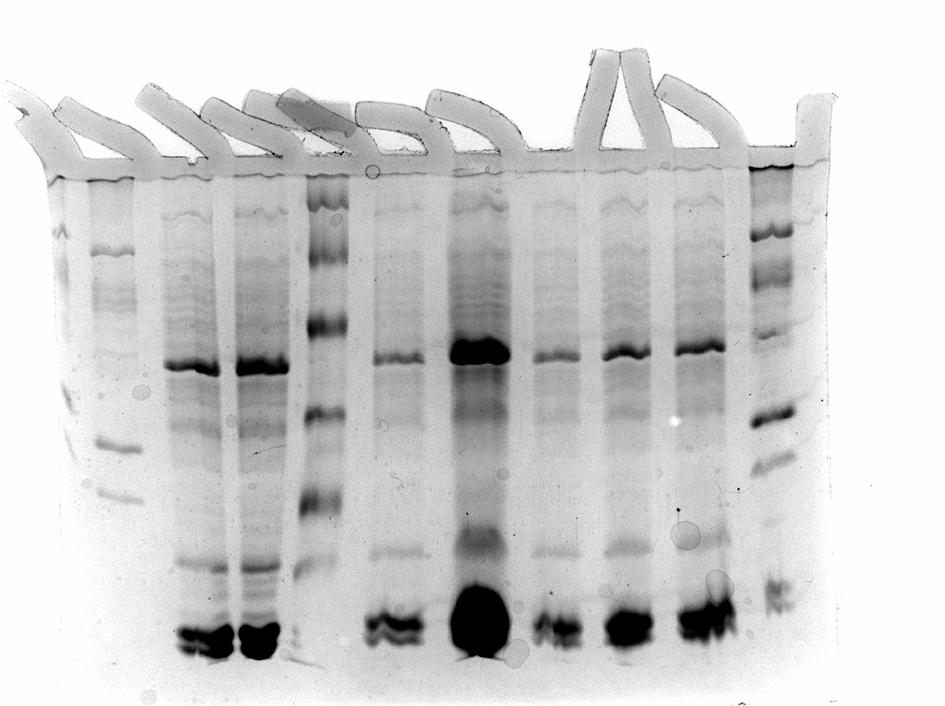

Supplement: Supplementary file 1 — Supplementary Material 1: Figure of the whole polyacrylamide gel with transglutaminase enzyme fractions. (PNG 378 KB) [file 253_2025_13606_Fig5_ESM.png]

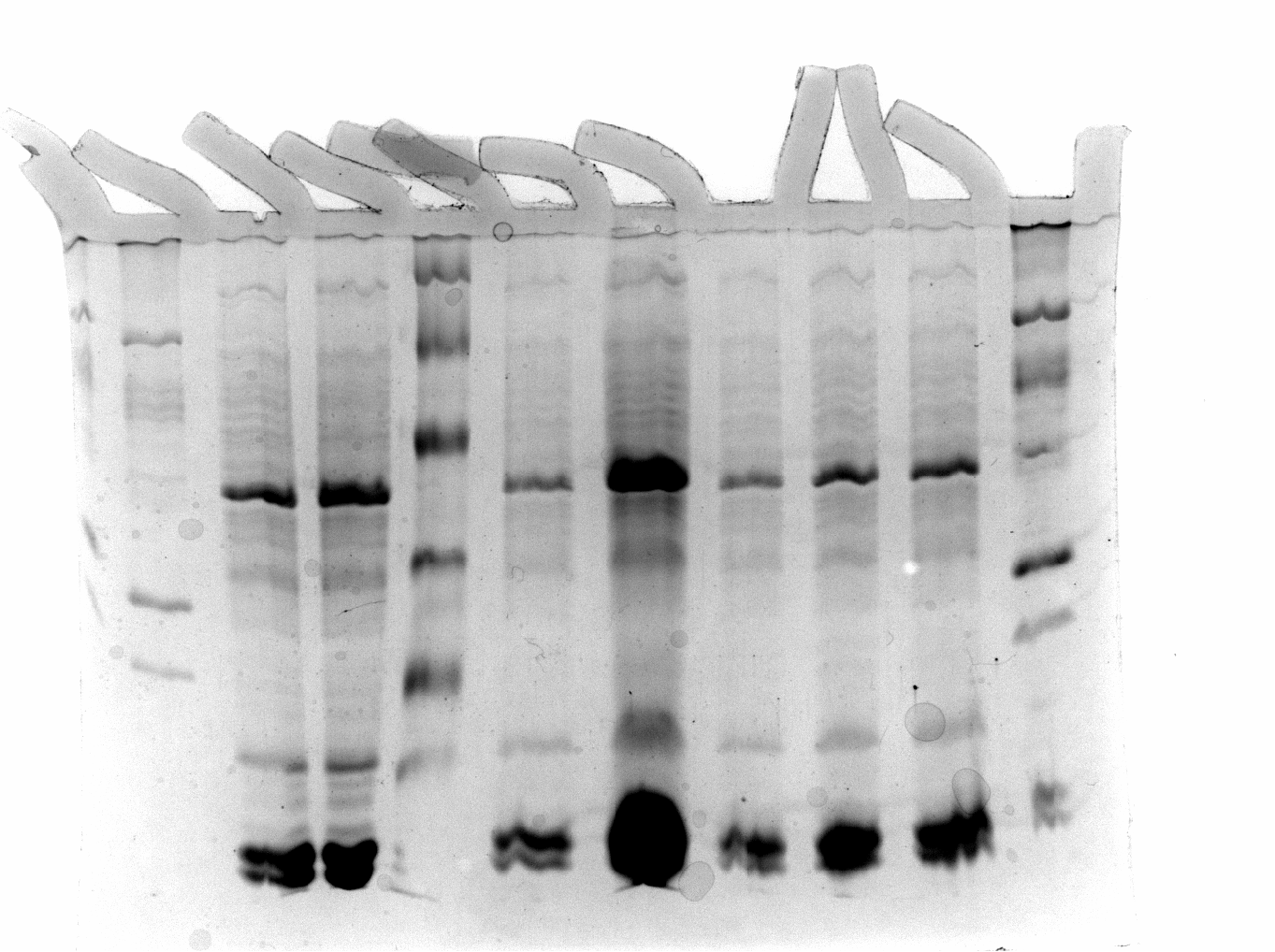

Supplement: Supplementary file 2 — High Resolution Image (TIF 3.96 MB) [file 253_2025_13606_MOESM1_ESM.tif]
